# Association between Neck Circumference and Subclinical Atherosclerosis among Chinese Steelworkers: A Cross-Sectional Survey

**DOI:** 10.3390/ijerph19116740

**Published:** 2022-05-31

**Authors:** Miao Yu, Lihua Wang, Shengkui Zhang, Hongman Feng, Jianhui Wu, Xiaoming Li, Juxiang Yuan

**Affiliations:** Department of Epidemiology and Health Statistics, School of Public Health, North China University of Science and Technology, Tangshan 063210, China; yumiao@stu.ncst.edu.cn (M.Y.); wanglihua@stu.ncst.edu.cn (L.W.); zhangsk@stu.ncst.edu.cn (S.Z.); fenghongman@stu.ncst.edu.cn (H.F.); wujianhui555@ncst.edu.cn (J.W.); lixiaoming@ncst.edu.cn (X.L.)

**Keywords:** neck circumference, carotid intima-media thickness, subclinical atherosclerosis, steelworkers

## Abstract

The purpose of this study was to determine whether neck circumference (NC) is associated with subclinical atherosclerosis among Chinese steelworkers in North China. A cross-sectional survey was conducted among steelworkers in northern China (*n* = 3467). Carotid intima-media thickness (CIMT) was measured at the distal wall of the common carotid artery proximal to the bifurcation point along a plaque-free segment 10 mm long on each side by B-ultrasound. The mean of the common CIMT was used bilaterally in this study. In the cross-sectional analysis, large NC was associated with the presence of abnormal CIMT. Logistic regression analysis was used to assess the relationship between NC tertiles and CIMT. The multivariable-adjusted odds ratio was 1.76 (95% CI: 1.40 to 2.22; *p* for trend <0.001) for the highest tertile versus the lowest tertile and was 1.07 (95% CI: 1.04 to 1.10; *p* < 0.001) per 1 standard deviation increment in NC. Among steelworkers in North China, relatively large NC level is associated with elevated odds of subclinical atherosclerosis.

## 1. Introduction

Cardiovascular disease (CVD) is the leading cause of death in China, accounting for 40% of deaths in the Chinese population [1]. About 2.4 million people in China died of atherosclerotic CVD (ASCVD) in 2016, accounting for 61% of deaths from CVD and 25% of all deaths [2]. Hence, early detection of atherosclerosis should be considered a top priority for CVD prevention in China. Carotid intima-media thickness (CIMT) is a common evaluation indicator of atherosclerosis that provides evidence of vascular disease before the onset of symptoms and is often used to evaluate the extent of early atherosclerosis (i.e., subclinical atherosclerosis) [3,4].

Obesity is associated with risk factors for CVD such as dyslipidemia and hypertension, promoting the development of atherosclerosis [5]. However, there is considerable variation in fat distribution among individuals with the same body mass index (BMI) status, leading to different metabolic risks, especially for upper body adiposity [6,7,8]. The underlying mechanism may be the release of excess free fatty acids from upper body adipose tissue, leading to triglyceride synthesis and ectopic deposition, and the resulting inflammation-mediated vascular damage that accelerates the onset of atherosclerosis [9,10,11,12].

Neck circumference (NC) is considered a relatively easy anthropometric measure to predict upper body fat distribution [13,14]. Compared with BMI or waist circumference (WC), NC has been suggested as a new measurement for assessing obesity because the effects of respiration, clothes, and postprandial abdominal distension are avoided. The limitations of BMI in measuring the distribution of fat are well established, as it only reflects the total weight of the human body, and the ratio of muscle fat, bone, or fluid is unable to be distinguished [14]. In certain populations, such as heavy manual workers, the increase in BMI is not directly related to their health status due to the increased muscle mass and weight. As a portable and time-saving anthropometric method, NC was independently correlated with visceral fat content and has been proven as the prediction of cardiometabolic risk [15]. However, the exact mechanism of the association between NC and CIMT is not fully understood, but the accumulation of adipose tissue in the upper body as an important risk factor for the aggregation of metabolic disorders and increased cardiovascular risk has been extensively studied [16].

Therefore, the NC as an indicator of upper body adiposity is beginning to show diagnostic value in evaluating atherosclerosis. To the best of our knowledge, the results of previous studies on NC and atherosclerosis were inconsistent [17,18]. Additionally, studies evaluating fat accumulation in the neck (as a proxy for subcutaneous fat in the upper body) and its association with CIMT from occupational-based population are still lacking. The present study aimed to evaluate the relationship between NC and CIMT among Chinese steelworkers.

## 2. Materials and Methods

### 2.1. Study Design and Population

This cross-sectional study reported the results of a baseline survey of steelworkers in 11 steel production sectors of HBIS Group’s Tangsteel Company in North China. All employees of the company underwent statutory annual physical examination from February to June 2017, and a total of 7661 participants were recruited. Overall, 4084 workers voluntarily completed carotid ultrasound examinations. After excluding 297 workers with incomplete questionnaire information and 320 workers with incomplete body measurement index data, a total of 3467 workers were included in the final analysis. The age ranges were 23 to 60 for male workers and 28 to 60 for female workers. The study was approved by the ethics committee of the North China University of Science and Technology (Ethic ID: 16040). Written informed consent was obtained from all participants.

### 2.2. Anthropometric Measurements

NC, WC, and hip circumference (HC) were measured three times by a plastic belt, and the average value of the measurement was taken. NC was measured horizontally from the midway of the neck, between the middle of the cervical spine and the middle of the anterior neck, with the head erect and eyes facing forward. The WC was measured at the midpoint between the lower edge of the rib and the upper edge of the iliac crest on the mid axillary line. HC was measured at the level of the maximum posterior extension of the hip in the horizontal plane. To avoid measurement bias, there was only one staff assigned to measure NC, WC, and HC all through the survey. The height and weight data that were ultimately used for analysis were accurate at 0.1 cm and 0.1 kg. BMI was defined as body weight (kg) divided by the square of the body height (m^2^). WHR was calculated as WC (cm) divided by HC (cm). BMI ≥ 25 kg/m^2^ [19] was defined as overweight and obesity. Abdominal obesity (AO) was defined as male WC ≥ 90 cm or female WC ≥ 80 cm [20].

### 2.3. Assessments of CIMT

A large number of studies have shown that CIMT evaluated by B-ultrasound is a marker of atherosclerosis in the arterial system. Therefore, subclinical atherosclerosis was able to be expressed by CIMT of common carotid artery (CCA) [21,22,23]. CIMT was measured at the distal wall of the CCA proximal to the bifurcation point along a plaque-free segment 10 mm long on each side. The mean of the common CIMT was used bilaterally in this study.

Carotid artery ultrasound scanning was carried out using a high-resolution B-mode topographic ultrasound system (PHILIPS, HD7, Shanghai, China). It was conducted by two trained ultrasound doctors who were blinded to clinical presentation and laboratory data. According to the 2007 European Society of Hypertension/European Society of Cardiology hypertension guideline recommends, we defined a CIMT > 0.9 mm as an abnormal CIMT [24].

### 2.4. Assessment of Covariates

Covariates mainly included age, sex, educational level, smoking status, drinking status, physical activity, dietary approaches to stop hypertension (DASH) score [25], dyslipidemia, diabetes, and elevated blood pressure.

### 2.5. Statistical Analysis

The measurement data were statistically described using mean and standard deviation if the normal distribution was followed. Student’s *t*-test or analysis of variance (ANOVA) were used for comparison among groups. Otherwise, the median (upper quartile–lower quartile) and Kruskal–Wallis test were used to describe and compare non-normal distribution continuous variables. The classification data were presented as numbers and percentages, and the *χ*^2^ test method was used for comparison among groups. Pearson correlation analysis was used to calculate the correlation coefficient between WC, NC, and metabolic parameters. Logistic regression analysis was used to assess the association between NC and CIMT. NC was included in the logistic regression analysis as a categorical variable divided into three groups by tertiles and as a continuous variable. Model 1 was unadjusted. Model 2 was adjusted for age and sex. Model 3 further adjusted education level, smoking status, drinking status, physical activity, DASH score, diabetes, dyslipidemia, and elevated blood pressure. We conducted a subgroup analysis by BMI, AO, sex, and age, to examine whether the relationship between NC and CIMT is reliable in the presence of potential confounding factors. All data were analyzed using SAS V.9.4 (SAS Institute, Cary, NC, USA) and MedCalc 19.6.0 (MedCalc Software, Ostend, Belgium). A two-tailed *p* < 0.05 was considered statistically significant.

## 3. Results

### 3.1. General Characteristics of the Participants

The demographic, clinical, and lifestyle characteristics of the participants according to CIMT status are shown in Table 1. A total of 3467 participants were included with a mean age of (46.01 ± 7.87) years. Among all workers included, the prevalence of abnormal CIMT (>0.9 mm) was 20.8%, while the prevalence of abnormal CIMT in male workers (22.03%) was significantly higher than that in female workers (9.06%) (Supplementary Table S1). Workers with abnormal CIMT had higher blood pressure, fasting blood glucose (FBG), TC, TG, LDL, BMI, WHR, and WC levels. There were no statistical differences in CIMT status in DASH Scores, physical activity, and HDL levels. As presented in Supplementary Table S2, larger NC was observed in workers with abnormal CIMT than those without (39.44 ± 3.08 cm vs. 38.44 ± 3.28 cm, *p* < 0.001). Supplementary Table S1 shows the basic characteristics of the participants according to sex. In addition, when grouped by tertiles of NC, the prevalence of abnormal CIMT raised obviously from the lowest tertile to the highest tertile group (14.46%, 21.19%, 26.70%, respectively) (Supplementary Table S2).

### 3.2. Relationship between WC, NC, and CIMT

The results of the Pearson correlation depicted that WC and NC was significantly correlated with systolic blood pressure (SBP), diastolic blood pressure (DBP), FBG, total cholesterol (TC), triglycerides (TG), low-density lipoprotein (LDL). The association was still significant (*p* < 0.05) even after adjusting for age, sex, educational level, smoking status, drinking status, physical activity, DASH score (Table 2).

We used logistic regression analysis to assess the relationship between NC and CIMT, with the lowest NC tertile group as a reference. As it is shown in Table 3, the odds for abnormal CIMT were higher with increasing NC tertiles. Compared with the lowest NC tertile, odds ratios (OR) of abnormal CIMT were observed to have significantly increased in the highest NC tertile (OR = 1.93; 95% confidence interval (CI): 1.54 to 2.41; *p* for trend <0.001) after adjusting for age and sex (Model 2). After adjustment for educational level, smoking status, drinking status, physical activity, and DASH score, dyslipidemia, elevated blood pressure, and diabetes only slightly reduced the magnitude of the odds for abnormal CIMT (OR = 1.76; 95% CI: 1.40 to 2.22; *p* for trend <0.001) (Model 3). The overall OR (95% CI) of abnormal CIMT was 1.07 (1.04 to 1.10) per 1-SD increment of NC when NC was considered as a continuous variable (Table 4).

The associations between NC tertiles and the odds for abnormal CIMT stratified by BMI, AO, sex, and age are presented in Table 5. Stratified analyses in BMI categories revealed a significant association between the highest NC tertile and abnormal CIMT in the group with BMI ≥ 25 kg/m^2^ (OR = 1.94; 95% CI: 1.37 to 2.76). Analyses stratified by sex indicated that there were significant associations between the highest NC tertile and abnormal CIMT in male (OR = 1.73; 95% CI: 1.36 to 2.19) and female (OR = 1.39; 95% CI: 0.98 to 4.70). As for age, in age 50 to 60, compared with those the lowest NC tertile, the highest NC tertile had increased odds of abnormal CIMT (OR = 1.97; 95% CI: 1.43 to 2.69). Each of the variables were observed with no interaction (all *p* for interaction >0.05).

Subsequently, by constructing the areas under ROC curves (AUCs), the predictive value of NC and other body measurement indexes for abnormal CIMT was evaluated. Considering that there are various age and sex groups, we assessed the AUCs of NC and other body measurement indexes for CIMT by age and sex groups. For sex groups, the AUCs of NC were 0.564 in male and 0.630 in female. The AUCs in male and female were the highest in WC (0.576) and WHR (0.679), respectively. For age groups, the age group of 23 to 39 had the highest AUCs value of NC both in male and female (male: 0.579; female: 0.709) (Supplementary Table S4).

Considering the main occupational hazards exposed to workers in steel enterprises such as dust, high temperature, noise, and carbon monoxide (see supplementary materials), we further adjusted these exposures on the basis of multivariate analysis and the results remained robust (Supplementary Table S3).

## 4. Discussion

Rare studies have explored the potential value of NC as a predictor of abnormal CIMT among the occupational population in China. In this cross-sectional study of steelworkers, the prevalence of abnormal CIMT was 20.8%, and a positive association was observed between relatively large NC and CIMT. In addition, similar results were obtained by sensitivity analysis and the association remained robust after further adjustments for occupational hazards, such as dust, high temperature, noise, and carbon monoxide. Our research shows that higher neck fat accumulation was also associated with metabolic dysregulation, characterized by elevated blood pressure and fasting blood glucose. Overall, these findings suggest that the excessive accumulation of neck fat in steelworkers was of great significance for predicting the prevalence of subclinical atherosclerosis.

Previous studies have reported that central obesity, hypertension, hypertr-iglyceridaemia, and impaired fasting glucose were associated with abnormal CIMT [26]. In our study, as with WC, NC was significantly associated with blood pressure, blood glucose, triglycerides, total cholesterol, and LDL, in line with previous studies [27,28]. Furthermore, the positive association between NC and the prevalence of abnormal CIMT remained robust after the control of metabolism-related indicators. Our strong evidence indicates that the relationship between NC and the prevalence of abnormal CIMT, even among steelworkers, might validate their results and provide valuable insights for further research. Previous studies have demonstrated the value of NC as an indicator of CIMT. In a cross-sectional study of 3274 offspring of Framingham heart study participants, Rosenquist et al. found that NC was an independent determinant of CIMT after adjusting for age, sex, blood pressure, diabetes, TC-to-HDL cholesterol ratio, and smoking [29]. However, Yashashwi et al. [18] examined the relationship between NC and subclinical atherosclerosis (assessed as coronary artery calcium and Carotid artery plaque) in retired National Football League (NFL) players and found no significant association between NC and subclinical atherosclerosis. Heterogeneity of the sample population and outcome assessment may lead to differences in research results. Sex stratified analysis provided further information that there was a sex difference in the correlation between NC and CIMT, and a positive correlation was found only in male subjects. This may be related to fewer female participants and the protection of estrogen in females. Epidemiological studies have shown that the prevalence and incidence of abnormal CMIT are influenced by sex and menopause. In men, CIMT progression is more correlated with traditional risk factors [30]. In our study, the number of female employees is relatively small. Therefore, the association between NC and atherosclerotic processes in females should be further explored in large-scale prospective studies.

To evaluate the predictive value of NC and other body composition indicators for CIMT, ROC analysis was used to calculate AUCs, and the results showed that NC has a low AUC value and may not be a good predictor of CIMT. However, none of the current studies provide data disaggregated by age group. Our results showed that, in the 50–60 age group, NC had the highest predictive value in comparison with other body measurement indexes in both sex groups. Similarly, stratified analysis of age was observed, with the highest tertile of NC positively associated with abnormal CIMT in the 50–60 age group. Previous research reported that age is an independent risk factor for CIMT progression, and older age is associated with a higher CIMT [31]. Moreover, the fat in the neck redistributes with age, and the middle of the neck was distributed with more fat, resulting in a relatively larger NC [32]. In our study, participants in the 50–60 age group had the highest number of abnormal CIMT, accounting for more than half. However, this rate was less than 10% in the 23–39 age group. Therefore, the larger NC and sufficient number of participants with abnormalities may highlight the role of NC in predicting abnormal CIMT.

It is well established that obesity is commonly known to be a heterogeneous disease. Differences in body fat distribution result in specific cardiovascular disease [33]. Previous studies have demonstrated that atherosclerosis is associated with endothelial dysfunction caused by excessive visceral adipose tissue (VAT) and perivascular adipose tissue (PVAT) [5,34]. VAT has been shown to be a major contributor to the release of free fatty acids throughout the body, particularly in obese individuals [8]. As an indicator of upper body obesity, NC might be a proxy for VAT [35]. The following mechanisms may explain the association between NC and CIMT. Firstly, adipose tissue not only stores fat but also secretes various bioactive adipokines [9]. The expansion of visceral adipose tissue increases the release of free fatty acids [36]. Increased free fatty acids are involved in endothelial dysfunction by altering adipokine expression, causing proliferation and migration of leukocytes [37]. Secondly, neck fat encompasses carotid arteries, an important PVAT depot. With the accumulation of fat, excess PVAT in the carotid arteries leads to chronic inflammation through infiltration of macrophages, oxidative stress, and chronic inflammation, which ultimately triggers vascular damage and development of proliferative vascular disease [16,34,38]. The excess free fatty acids and PVAT might be a potential link between NC and CIMT. Therefore, NC could serve as a predictive indicator for the future development of CIMT, and more research is needed, especially prospective studies to further understand the relationship mechanism between NC and CIMT.

The limitations of our study should be noted. First, the nature of the cross-sectional study limited our ability to infer causal relationships between NC and CIMT to some extent. Second, we used NC to assess subcutaneous fat in the neck, but we cannot quantify the fat and muscle content. Therefore, the proportions of fat and muscle are unknown. Third, our data are based on steelworkers, most of whom are male in northern China, so the ability to generalize the conclusions to the general population is limited. Fourth, compared with workers who did not take carotid ultrasound, those who did were older, less were male, and they had higher SBP, DBP, and FBG levels (Supplementary Table S5). These workers might be more concerned about their own physical condition due to age and poor health. This introduces volunteer bias. Nevertheless, the detailed data and large sample size we collected can provide valuable information for screening CIMT in the occupational physical examination.

## 5. Conclusions

In conclusion, large NC is associated with elevated odds of subclinical atherosclerosis in steelworkers. Measurement of NC may provide a more complete understanding of subclinical atherosclerosis risk with variation in fat distribution.

## Figures and Tables

**Table 1:** Basic characteristics of participants according to CIMT status.

Variables	Total	Normal CIMT	Abnormal CIMT	*p* Value
	*n* = 3467	*n* = 2746	*n* = 721	
Sex (male), *n* (%)	3136 (90.45)	2245 (89.04)	691 (95.84)	<0.001
Age (years), mean (SD)	46.01 (7.87)	44.91 (7.96)	50.19 (5.87)	<0.001
SBP (mmHg), mean (SD)	129.53 (16.53)	128.30 (16.12)	134.1 (17.27)	<0.001
DBP (mmHg), mean (SD)	82.80 (10.62)	82.23 (10.44)	84.98 (10.99)	<0.001
FBG (mmol/L), mean (SD)	6.13 (1.39)	6.06 (1.33)	6.40 (1.58)	<0.001
TC (mmol/L), mean (SD)	5.15 (0.98)	5.08 (0.96)	5.42 (1.02)	<0.001
TG (mmol/L), median (IQR)	1.29 (0.89–1.94)	1.26 (0.87–1.93)	1.37 (0.95–1.97)	0.003
HDL (mmol/L), mean (SD)	1.31 (0.33)	1.31 (0.33)	1.29 (0.33)	0.225
LDL (mmol/L), mean (SD)	3.25 (0.87)	3.19 (0.85)	3.49 (0.91)	<0.001
BMI (kg/m^2^), mean (SD)	25.21 (3.29)	25.09 (3.29)	25.65 (3.27)	<0.001
WC (cm), mean (SD)	89.42 (9.75)	88.77 (9.80)	91.89 (9.14)	<0.001
WHR, mean (SD)	0.88 (0.06)	0.88 (0.06)	0.90 (0.06)	<0.001
NC (cm), mean (SD)	38.65 (3.27)	38.44 (3.28)	39.44 (3.08)	<0.001
DASH score, mean (SD)	21.59 (2.38)	21.60 (2.37)	21.55 (2.42)	0.614
Age (years), *n* (%)				<0.001
23–39	726 (20.94)	682 (24.84)	44 (6.10)	
40–49	1425 (41.10)	1182 (43.04)	243 (33.70)	
50–60	1316 (37.96)	882 (32.12)	434 (60.19)	
Education level, *n* (%)				<0.001
Primary or Middle	1021 (29.45)	735 (26.77)	286 (39.67)	
High school or college	1827 (52.70)	1453 (52.91)	374 (52.87)	
University and above	619 (17.85)	558 (20.32)	61 (8.46)	
Smoking status, *n* (%)				<0.001
Never	1435 (41.39)	1197 (43.59)	238 (33.01)	
Ever	230 (6.63)	168 (6.12)	62 (8.06)	
Current	1802 (51.98)	1381 (50.29)	421 (58.39)	
Drinking status, *n* (%)				<0.001
Never	2023 (58.35)	1644 (59.87)	379 (52.57)	
Ever	116 (3.35)	81 (2.95)	35 (4.85)	
Current	1328 (38.30)	1021 (37.18)	307 (42.58)	
BMI (kg/m^2^), *n* (%)				<0.001
<25	1283 (37.01)	1062 (38.67)	221 (30.65)	
25–29	1561 (45.02)	1216 (44.28)	345 (47.85)	
≥30	623 (17.97)	468 (17.04)	155 (21.5)	
Physical activity, *n* (%)				0.978
Low	37 (1.07)	29 (1.06)	8 (1.11)	
Moderate	245 (7.07)	193 (7.03)	52 (7.21)	
High	3185 (91.87)	2524 (91.92)	661 (91.68)	

SBP, systolic blood pressure; DBP, diastolic blood pressure; FBG, fasting blood glucose, TC, total cholesterol; TG, triglycerides; HDL, high-density lipoprotein; LDL, low-density lipoprotein.

**Table 2:** Correlations between WC, NC, and metabolic characteristics.

Variables	Waist Circumference		Neck Circumference	
	*β*	*p* Value	*β*	*p* Value
SBP	0.248	<0.001	0.217	<0.001
DBP	0.165	<0.001	0.165	<0.001
FBG	0.156	<0.001	0.156	<0.001
TC	0.121	<0.001	0.071	<0.001
TG	0.193	<0.001	0.171	<0.001
LDL-C	0.139	<0.001	0.094	<0.001

All models were adjusted for age (continuous variable), sex (male, female), educational level (primary or middle, high school or college, university and above), smoking status (no/yes), drinking status (no/yes), physical activity (low, moderate, high), DASH score (continuous variable).

**Table 3:** Presence of abnormal CIMT in relation to NC tertiles.

Atherosclerosis	Neck Circumference			
	T1 (*n* = 1155)	T2 (*n* = 1146)	T3 (*n* = 1165)	*p* for Trend
Model 1	1.00	1.59 (1.28 to 1.98)	2.15 (1.75 to 2.66)	<0.001
Model 2	1.00	1.36 (1.08 to 1.72)	1.93 (1.54 to 2.41)	<0.001
Model 3	1.00	1.32 (1.04 to 1.65)	1.76 (1.40 to 2.22)	<0.001

Model 1 unadjusted. Model 2 was further adjusted for age (continuous variable) and sex (male, female). Model 3 was further adjusted for educational level (primary or middle, high school or college, university and above), smoking status (no/yes), drinking status (no/yes), physical activity (low, moderate, high), DASH score (continuous variable), dyslipidemia (no/yes), elevated blood pressure (no/yes), diabetes (no/yes).

**Table 4:** Presence of abnormal CIMT in relation to NC.

Atherosclerosis	Per 1 SD, as Continuous Variable	*p* Value
	OR (95% CI)	
Model 1	1.10 (1.07 to 1.13)	<0.001
Model 2	1.08 (1.05 to 1.12)	<0.001
Model 3	1.07 (1.04 to 1.10)	<0.001

Model 1 unadjusted. Model 2 was further adjusted for age (continuous variable), sex (male, female). Model 3 was further adjusted for educational level (primary or middle, high school or college, university and above), smoking status (no/yes), drinking status (no/yes), physical activity (low, moderate, high), DASH score (continuous variable), dyslipidemia (no/yes), elevated blood pressure (no/yes), diabetes (no/yes).

**Table 5:** Multivariate adjusted odds ratios for the association between NC tertiles and abnormal CIMT, stratified by BMI, abdominal obesity, sex, and age.

Groups	Neck Circumference			
	T1 (*n* = 1155)	T2 (*n* = 1146)	T3 (*n* = 1165)	*p* for Interaction
BMI				0.484
<25 kg/m^2^	1.00	1.20 (0.85 to 1.69)	1.28 (0.79 to 2.08)	
≥25 kg/m^2^	1.00	1.44 (1.00 to 2.06)	1.94 (1.37 to 2.76)	
Abdominal obesity				0.135
no	1.00	1.46 (1.09 to 1.95)	1.48 (0.94 to 2.33)	
yes	1.00	0.80 (0.52 to 1.24)	1.16 (0.76 to 1.76)	
Sex				0.131
Male	1.00	1.29 (1.02 to 1.64)	1.73 (1.36 to 2.19)	
Female	1.00	1.24 (0.38 to 4.06)	1.39 (0.98 to 4.70)	
Age				0.060
23–39	1.00	1.15 (0.48 to 2.73)	1.63 (0.72 to 3.68)	
40–49	1.00	1.30 (0.89 to 1.91)	1.43 (0.98 to 2.09)	
50–60	1.00	1.30 (0.95 to 1.77)	1.97 (1.43 to 2.69)	

*p* for interaction were estimated using a log likelihood ratio test to compare models with and without cross-product interaction terms and were derived by NC × characteristics. Adjusted for age (continuous variable), sex (male, female), educational level (primary or middle, high school or college, university and above), smoking status (no/yes), drinking status (no/yes), physical activity (low, moderate, high), DASH score (continuous variable), dyslipidemia (no/yes), elevated blood pressure (no/yes), diabetes (no/yes).

## Data Availability

The datasets generated and analyzed in the course of this study are available from the corresponding author on reasonable request.

## Supplementary Materials

The following supporting information can be downloaded at: https://www.mdpi.com/article/10.3390/ijerph19116740/s1, Table S1: Basic characteristics of participants according to sex; Table S2: Basic characteristics of participants according to NC; Table S3: Association between NC and abnormal CIMT after further adjusted for the main occupational hazards. Table S4: Sex and age-specific areas under the receiver operating characteristic curves in the steelworkers. Table S5: Comparison of characteristics of workers who participated and did not participate in carotid ultrasound.
