# Supplementary material for: Association between Neck Circumference and Subclinical Atherosclerosis among Chinese Steelworkers: A Cross-Sectional Survey
Source: Int J Environ Res Public Health. 2022 May 31;19(11):6740. doi: 10.3390/ijerph19116740 (PMC9180598; doi:10.3390/ijerph19116740)
Supplement: Supplementary file 1 [file ijerph-19-06740-s001.zip › ijerph-1701966-supplementary.pdf]

Supplementary file

Association between neck circumference and subclinical atherosclerosis among Chinese steelworkers: a cross-sectional survey

Assessment of covariates

The level of education was divided into three categories: "primary or middle", "high school or college", and "university and above".

Standard study protocols were used to train qualified physicians and nurses prior to this survey. Height and weight were measured three times each. The participants stood upright and barefoot in light clothes.

Blood pressure measurements were performed three times five-minute intervals using an electronic sphygmomanometer (OMRON, HBP-1100, China), and the participants were required to rest for more than ten minutes. Finally, the mean was obtained for analysis. Elevated blood pressure was defined as current systolic blood pressure ≥140 mmHg, or diastolic blood pressure ≥90 mmHg, or if the patient was receiving antihypertensive therapy[1].

Participants' anterior elbow vein blood was collected and centrifuged at room temperature (3000 r/min, 15 minutes) immediately. All blood samples were tested in the central laboratory of Tangshan Hongci Hospital Laboratory using automatic biochemical analysers (Mindrary, BS-800, China) within four hours. Total cholesterol (TC) ≥6.22 mmol/L or low-density lipoprotein (LDL) ≥4.11 mmol/L or high-density lipoprotein (HDL) ≤1.04 mmol/L or triglycerides (TG) ≥2.32 mmol/L, or patients undergoing lipid-lowering therapy were considered to demonstrate dyslipidaemia[2]. Diabetes was defined as fasting blood glucose ≥7.0 mmol/L or if the patient was receiving hypoglycemic therapy[3].

Smoking status was evaluated from self-reported information, mainly including the age at starting smoking and the number of cigarettes consumed per day, and was divided into never smokers (who had never smoked in their lifetime); ever smokers (who had quit cigarettes earlier than 12 months before) and current smokers (who had regularly consumed ≥1 cigarette/day for at least the past 12 months)[4].

Drinking status was evaluated from self-reported information, mainly including the amount and frequency of alcohol consumed per week and was divided into never drinkers (never or almost never drank alcohol in the past 12 months and had not drunk in most weeks in any past year); ever drinkers (did not drink alcohol in most weeks in the past 12 months but did so in some past year(s)) and current drinkers (drank alcohol usually at least once a week over the past 12 months)[5].

Dietary patterns were assessed based on the DASH diet score[6]. Dietary patterns were assessed based on the DASH diet score, which was based on eight foods and nutrients that were either emphasized or deemphasized in the DASH-style diet[7]. Each component was scored from 1 to 5 points according to fifths of intake, with 5 being the best score for higher intake of vegetables, fruits, nuts and legumes, whole grains, and low fat dairy products and for lower intake of sugar sweetened drinks, red and processed meats, and sodium.

The calculation of metabolic equivalents was based on the International Physical Activity Questionnaire (IPAQ)[8]. The workers with metabolic equivalent task (MET) [min/week] values < 600, 600-3000 and > 3000 were classified as having a low, moderate, and high level of physical activity respectively.

Participants were required to fast overnight before the health examination and blood collection. Participants' anterior elbow vein blood was collected and centrifuged at room temperature (3000 r/min, 15 minutes) immediately. All blood samples were tested in the central laboratory of Tangshan Hongci Hospital Laboratory using automatic biochemical analysers (mindrary, BS-800, China) within four hours.

Assessment of main occupational hazards

Exposure to dust was defined as workers who may be exposed to productive dust (inorganic dust, organic dust or mixed dust) during production (GBZ/T 229.1–2010). The total dust in the air of workplace was collected at the breathing zone with a filter membrane, and it's concentration was calculated based on the increased weight of the filter membrane and the amount of gas collected. When the dust concentration in the air ≤50 mg/m^3, a filter membrane with a diameter of 37mm or 40mm was used, otherwise a filter membrane with a diameter of 75mm would be used (GBZ/T 192.1–2007)[9].

Exposure to high temperature (heat stress work) was defined as the average wet-bulb globe temperature (WBGT) index of the workplace is equal or greater than 25℃ in the process of production (GBZ 2.2–2007)[10]. The WBGT index was measured by black-wet bulb globe thermometer. If there was no productive heat source in the workplace, three measuring points were selected to take the average value of WBGT index, while where there was a productive heat source, 3 to 5 measuring points were selected to take the average value of WBGT index. If the workplace was isolated into different thermal or ventilated environment, 2 measuring points were selected to take the average value of WBGT index (GBZ/T 189.7–2007)[11].

Exposure to industrial toxicant was defined as workers who may be exposed to a variety of harmful chemicals (the toxicant specifically refers to carbon monoxide in this population) during production (GBZ/T 229.2–2010)[12]. Carbon monoxide or carbon dioxide in the air of workplace was pumped into the Non-Dispersive Infrared-Ray (NDIR) analyzer and selectively absorbs their infrared rays. The concentration of carbon monoxide was determined according to the absorption value (GBZ/T 160.28–2004)[13].

Exposure to noise was defined as workers who exposed to a noisy environment where the 8h/d or 40h/week equivalent A-weighted sound pressure level is ≥80dB, which may be harmful to health and hearing (GBZ/T 229.4–2012)[14]. The workplace production noise was measured by a sound level meter. If the distribution of sound field in the workplace was uniform (between-field difference of A-sound levels were less than 3dB(A)), three measuring points were selected to take the average value, otherwise it should be divided into several sound level areas. In each sound field, two measuring points were selected to take the average value (GBZ/T 189.8–2007)[15].

Table of contents

Supplementary Table S1 Basic characteristics of participants according to sex

Variables	Total	Male	Female	*P* value
	n=3467	*n*=3136	*n*=331	
Age (years), mean (SD)	46.01 (7.87)	46.14 (8.08)	44.72 (44.14)	0.002
DASH score, mean (SD)	21.59 (2.37)	21.47 (2.34)	22.69 (2.46)	<0.001
BMI (kg/m^2), mean (SD)	25.21 (3.29)	25.36 (3.29)	23.75 (2.99)	<0.001
WC (cm), mean (SD)	89.42 (9.75)	90.19 (9.41)	82.12 (9.91)	<0.001
WHR, mean (SD)	0.88 (0.06)	0.89 (0.06)	0.83 (0.07)	<0.001
NC (cm), mean (SD)	38.65 (3.27)	39.05 (2.97)	34.81 (3.41)	<0.001
SBP (mmHg), mean (SD)	129.53 (16.53)	130.30 (16.53)	121.90 (14.51)	<0.001
DBP (mmHg), mean (SD)	82.80 (10.62)	83.34 (10.56)	77.72 (9.72)	<0.001
FBG (mmol/L), mean (SD)	6.13 (1.39)	6.17 (1.41)	5.76 (1.13)	<0.001
TC (mmol/L), mean (SD)	5.15 (0.98)	5.16 (0.98)	5.08 (0.97)	<0.001
TG (mmol/L), median (IQR)	1.29 (0.89–1.94)	1.34 (0.92–2.00)	0.91 (0.73–1.33)	<0.001
HDL-C (mmol/L), mean	1.31 (0.33)	1.29 (0.32)	1.52 (0.35)	<0.001
LDL-C (mmol/L), mean (SD)	3.25 (0.87)	3.26 (0.87)	3.16 (0.89)	0.045
Age (years), n (%)				<0.001
23–39	726 (20.94)	663 (21.14)	63 (19.03)	
40–49	1425 (41.10)	1201 (38.30)	224 (67.67)	
50–60	1316 (37.96)	1272 (40.56)	44 (13.29)	
Education level, n (%)				0.042
Primary or Middle	1021 (29.45)	943 (27.20)	78 (23.56)	
High school or college	1827 (52.70)	1641 (52.33)	186 (56.19)	
University and above	619 (17.85)	552 (17.60)	67 (20.24)	
Smoking status, n (%)				<0.001
Never	1435 (41.39)	1141 (36.38)	294 (88.82)	
Ever	230 (6.63)	224 (7.14)	6 (1.81)	
Current	1802 (51.98)	1771 (56.47)	31 (9.37)	
Drinking status, n (%)				<0.001
Never	2023 (58.35)	1720 (54.85)	303 (91.54)	
Ever	116 (3.35)	110 (3.51)	6 (1.81)	
Current	1328 (38.30)	1306 (41.65)	22 (6.65)	
Physical activity, n (%)				0.651
Low	37 (1.07)	35 (1.12)	2 (0.60)	
Moderate	245 (7.07)	220 (7.02)	25 (7.55)	
High	3185 (91.87)	2881 (91.87)	304 (91.84)	
BMI (kg/m^2), n (%)				<0.001
<25	1283 (37.01)	1090 (34.76)	193 (58.31)	
25–29	1561 (45.02)	1452 (46.30)	109 (32.93)	
≥30	623 (17.97)	594 (18.94)	29 (8.76)	
Abnormal CIMT, n (%)	721 (20.80)	691 (22.03)	30 (9.06)	<0.001

Supplementary Table S2 Basic characteristics of participants according to NC

Variables	Neck circumference			*P* value
	T1 (*n*=3467)	T2 (*n*=3136)	T3 (*n*=331)	
Age (years), mean (SD)	45.36 (7.94)	46.41 (7.88)	46.24 (7.76)	0.003
DASH score, mean (SD)	21.86 (2.43)	21.60 (2.28)	21.30 (2.40)	<0.001
BMI (kg/m^2), mean (SD)	23.12 (2.68)	25.01 (2.44)	27.47 (3.14)	<0.001
WC (cm), mean (SD)	81.80 (7.63)	88.99 (6.44)	97.39 (7.95)	<0.001
WHR, mean (SD)	0.85 (0.06)	0.89 (0.05)	0.91 (0.05)	<0.001
SBP (mmHg), mean (SD)	125.23 (15.82)	129.61 (16.14)	133.73 (16.52)	<0.001
DBP (mmHg), mean (SD)	80.38 (10.07)	83.41 (10.54)	84.61 (10.77)	<0.001
FBG (mmol/L), mean (SD)	5.88 (1.17)	6.11 (1.32)	6.40 (1.60)	<0.001
TC (mmol/L), mean (SD)	5.03 (0.94)	5.17 (0.98)	5.25 (1.01)	<0.001
TG (mmol/L), median (IQR)	1.02(0.75–1.50)	1.32 (0.93–1.95)	1.57 (1.09–2.28）	<0.001
HDL (mmol/L), mean (SD)	1.42 (0.35)	1.30 (0.32)	1.21 (0.27)	<0.001
LDL (mmol/L), mean (SD)	3.13 (0.82)	3.28 (0.86)	3.35 (0.90)	<0.001
Age (years), n (%)				<0.001
23–39	263 (22.77)	218 (19.01)	245 (21.03)	
40–49	520 (45.02)	449 (39.15)	456 (39.14)	
50–60	372 (32.21)	480 (41.85)	464 (39.83)	
Education level, n (%)				0.059
Primary or Middle	315 (27.27)	334 (29.12)	372 (31.93)	
High school or college	613 (53.07)	606 (52.83)	608 (52.19)	
University and above	227 (19.65)	207 (18.05)	185 (15.88)	
Smoking status, n (%)				<0.001
Never	579 (50.13)	468 (40.80)	388 (33.30)	
Ever	62 (5.37)	79 (6.89)	89 (7.64)	
Current	514 (44.50)	600 (52.31)	688 (59.06)	
Drinking status, n (%)				<0.001
Never	773 (66.93)	644 (56.15)	606 (52.02)	
Ever	31 (2.68)	45 (3.92)	40 (3.43)	
Current	351 (30.39)	458 (39.93)	519 (44.55)	
Physical activity, n (%)				0.486
Low	13 (1.13)	13 (1.13)	11 (0.94)	
Moderate	77 (6.67)	73 (6.36)	95 (8.15)	
High	1065 (92.21)	1061 (92.50)	1059 (90.90)	
BMI (kg/m^2), n (%)				<0.001
<25	763 (66.06)	383 (33.39)	137 (11.76)	
25–29	335 (29.00)	647 (56.41)	579 (49.70)	
≥30	57 (4.94)	117 (10.20)	449 (38.54)	
Abnormal CIMT, n (%)	167 (14.46)	243 (21.19)	311 (26.70)	<0.001

Supplementary Table S3 Association between NC and abnormal CIMT after further adjusted for the main occupational hazards

	Reference	OR (95% CI)	*P* value
Middle tertile versus lowest tertile	1.00	1.36 (1.07 to 1.72)	<0.001*
Highest tertile versus lowest tertile	1.00	176 (1.39 to 2.24)	
Per 1 SD, as continuous variable	1.00	1.05 (1.01 to 1.09)	<0.001#

* *P* for trend. # *P* value.

Adjusted for age (continuous variable), sex (male, female), educational attainment (primary or middle, high school or college, university and above), smoking status (no/yes), drinking status (no/yes), physical activity (low, moderate, high), DASH score (continuous variable), dyslipidemia (no/yes), hypertension (no/yes), diabetes (no/yes) and dust (no/yes), high temperature (no/yes), noise (no/yes), and carbon monoxide (no/yes) exposure in each exposure metric.

Supplementary Table S4 Sex and age-specific areas under the receiver operating characteristic curves in the steelworkers.

	**NC**			**WC**			**WHR**			**BMI**		
	AUC	95% CI		AUC	95% CI		AUC	95% CI		AUC	95% CI	
Male												
Overall	0.564	0.547	0.582	0.576	0.559	0.594	0.575	0.557	0.592	0.539	0.521	0.557
23–39	0.579	0.540	0.617	0.646	0.608	0.683	0.625	0.587	0.662	0.597	0.559	0.635
40–49	0.549	0.520	0.577	0.571	0.542	0.599	0.571	0.543	0.599	0.543	0.515	0.572
50–60	0.574	0.546	0.601	0.572	0.545	0.600	0.543	0.515	0.571	0.549	0.522	0.577
Female												
Overall	0.630	0.575	0.682	0.671	0.618	0.722	0.679	0.625	0.729	0.555	0.500	0.610
23–39	0.709	0.581	0.816	0.714	0.586	0.821	0.759	0.634	0.858	0.614	0.483	0.734
40–49	0.664	0.598	0.726	0.724	0.661	0.781	0.727	0.664	0.784	0.587	0.519	0.652
50–60	0.636	0.477	0.776	0.569	0.411	0.717	0.615	0.457	0.758	0.621	0.462	0.762

Supplementary Table S5 Comparison of characteristics of workers who participated and did not participate in carotid ultrasound

Variables, mean (SD)	No participated	Participated	*P* value
	n=4194	***n***=3467	
Age (years)	41.47 (8.68)	46.01 (7.87)	<0.001
BMI (kg/m^2)	25.62 (3.75)	25.21 (3.29)	0.655
Systolic blood pressure (mmHg)	128.40 (15.36)	129.53 (16.53)	0.004
Diastolic blood pressure (mmHg)	82.26 (10.12)	82.80 (10.62)	0.005
Fasting blood glucose (mmol/L)	6.04 (1.25)	6.13 (1.39)	0.003
Total cholesterol (mmol/L)	5.12 (0.95)	5.15 (0.98)	0.190
Triglycerides (mmol/L)	1.30 (0.90–1.95)	1.29 (0.89–1.94)	0.785
HDL-C (mmol/L)	1.30 (0.33)	1.31 (0.33)	0.162
LDL-C (mmol/L)	3.19 (0.84)	3.25 (0.87)	0.004
Variables, N (%)			
Sex (male)	3908(93.16)	3136 (90.45)	0.002

References

1. Wang Z, Chen Z, Zhang L, Wang X, Hao G, Zhang Z, Shao L, Tian Y, Dong Y, Zheng C *et al*: **Status of Hypertension in China: Results From the China Hypertension Survey, 2012-2015**. *Circulation* 2018, **137**(22):2344-2356.
2. revision Jcfg: **2016 Chinese guidelines for the management of dyslipidemia in adults**. *Journal of geriatric cardiology : JGC* 2018, **15**(1):1-29.
3. Yang SH, Dou KF, Song WJ: **Prevalence of diabetes among men and women in China**. *The New England journal of medicine* 2010, **362**(25):2425-2426; author reply 2426.
4. Li X, Cui S, Wu J, Wang L, Yuan J: **Job category differences in the prevalence and associated factors of insomnia in steel workers in China**. *International journal of occupational medicine and environmental health* 2020, **33**(2):215-233.
5. Millwood IY, Walters RG, Mei XW, Guo Y, Yang L, Bian Z, Bennett DA, Chen Y, Dong C, Hu R *et al*: **Conventional and genetic evidence on alcohol and vascular disease aetiology: a prospective study of 500 000 men and women in China**. *Lancet (London, England)* 2019, **393**(10183):1831-1842.
6. Maskarinec G, Lim U, Jacobs S, Monroe KR, Ernst T, Buchthal SD, Shepherd JA, Wilkens LR, Marchand LL, Boushey CJ: **Diet Quality in Midadulthood Predicts Visceral Adiposity and Liver Fatness in Older Ages: The Multiethnic Cohort Study**. *Obesity (Silver Spring, Md)* 2017, **25**(8):1442-1450.
7. Fung TT, Chiuve SE, McCullough ML, Rexrode KM, Logroscino G, Hu FB: **Adherence to a DASH-style diet and risk of coronary heart disease and stroke in women**. *Archives of internal medicine* 2008, **168**(7):713-720.
8. Celis-Morales CA, Perez-Bravo F, Ibañez L, Salas C, Bailey MES, Gill JMR: **Objective vs. self-reported physical activity and sedentary time: effects of measurement method on relationships with risk biomarkers**. *PloS one* 2012, **7**(5):e36345.
9. **Determination of dust in the air of workplace. Part 1: Total dust concentration.** [http://niohp.chinacdc.cn/zyysjk/zywsbzml/201210/t20121012_70522.htm]
10. **Occupational exposure limits for hazardous agents in the workplace. Part 2: Physical agents** [http://niohp.chinacdc.cn/zyysjk/zywsbzml/201303/t20130329_79199.htm]
11. **Measurement of physical agents in workplace. Part 7: Heat Stress** [http://niohp.chinacdc.cn/zyysjk/zywsbzml/201210/t20121012_70527.htm]
12. **Classification of occupational hazards at workplaces. Part 2: Occupational exposure to chemicals** [http://niohp.chinacdc.cn/zyysjk/zywsbzml/201210/t20121012_70489.htm]
13. **Methods for determination of inorganic carbon compounds in the air of workplace** [http://niohp.chinacdc.cn/zyysjk/zywsbzml/201210/t20121015_70624.htm]
14. **Classification of occupational hazards at workplaces. Part 4: Occupational exposure to noise** [http://niohp.chinacdc.cn/zyysjk/zywsbzml/201307/t20130715_84934.htm]
15. **Measurement of physical agents in workplace. Part 8: Noise** [http://niohp.chinacdc.cn/zyysjk/zywsbzml/201210/t20121012_70526.htm]
